# Interleukin-1 beta-induced up-regulation of opioid receptors in the untreated and morphine-desensitized U87 MG human astrocytoma cells

**DOI:** 10.1186/1742-2094-9-252

**Published:** 2012-11-20

**Authors:** Linda Staikos Byrne, Jinsong Peng, Sraboni Sarkar, Sulie L Chang

**Affiliations:** 1 Institute of NeuroImmune Pharmacology, Seton Hall University, 400 South Orange Ave, South Orange, NJ, 07079, USA; 2 Department of Biological Sciences, Seton Hall University, 400 South Orange Ave, South Orange, NJ, 07079, USA; 3 Wuhan Centers for Disease Control and Prevention, Wuhan, People’s Republic of China

**Keywords:** IL-1β, Morphine, Mu-opioid receptor, U87 MG astrocytoma cells

## Abstract

**Background:**

Interleukin-1beta (IL-1β) is a pro-inflammatory cytokine that can be produced in the central nervous system during inflammatory conditions. We have previously shown that IL-1β expression is altered in the rat brain during a morphine tolerant state, indicating that this cytokine may serve as a convergent point between the immune challenge and opiate mediated biological pathways. We hypothesized that IL-1β up-regulates opioid receptors in human astrocytes in both untreated and morphine-desensitized states.

**Methods:**

To test this hypothesis, we compared the basal expression of the mu (MOR), delta (DOR), and kappa (KOR) opioid receptors in the human U87 MG astrocytic cell line to SH-SY5Y neuronal and HL-60 immune cells using absolute quantitative real time RT-PCR (AQ-rt-RT-PCR). To demonstrate that IL-1β induced up-regulation of the MOR, DOR and KOR, U87 MG cells (2 x 10^5^ cells/well) were treated with IL-1β (20 ng/mL or 40 ng/mL), followed by co-treatment with interleukin-1 receptor antagonist protein (IL-1RAP) (400 ng/mL or 400 ng/mL). The above experiment was repeated in the cells desensitized with morphine, where U87 MG cells were pre-treated with 100 nM morphine. The functionality of the MOR in U87 MG cells was then demonstrated using morphine inhibition of forksolin-induced intracellular cAMP, as determined by radioimmunoassay.

**Results:**

U87 MG cells treated with IL-1β for 12 h showed a significant up-regulation of MOR and KOR. DOR expression was also elevated, although not significantly. Treatment with IL-1β also showed a significant up-regulation of the MOR in U87 MG cells desensitized with morphine. Co-treatment with IL-1β and interleukin-1 receptor antagonist protein (IL-1RAP) resulted in a significant decrease in IL-1β-mediated MOR up-regulation.

**Conclusion:**

Our results indicate that the pro-inflammatory cytokine, IL-1β, affects opiate-dependent pathways by up-regulating the expression of the MOR in both untreated and morphine-desensitized U87 MG.

## Background

In the brain, astrocytes are an important component of the blood–brain barrier and participate in the maintenance of homeostasis. They are also a main producer of cytokines and chemokines [1]. In addition, astrocytes are capable of surviving under inflammatory conditions and are resistant to death receptor-mediated apoptosis [2].

Interleukin-1 (IL-1) is a pro-inflammatory cytokine expressed in the central nervous system (CNS). It is produced by a wide variety of cells, such as glia, astrocytes, neurons, monocytes and endothelial cells [3,4]. During inflammation, IL-1 can activate the paraventricular nucleus of the hypothalamus, resulting in the release of corticotrophin-releasing hormone and subsequent activation of the hypothalamic-pituitary-adrenal (HPA) axis [4].

Three opioid receptors have been identified to date – mu (MOR), delta (DOR) and kappa (KOR). Morphine, which has a higher affinity for the MOR compared to the KOR and DOR [5-7], elicits its effects mainly through the MOR. Morphine is well known for its analgesic effects and addictive properties [8], as well as its ability to alter the endocrine and immune systems [9,10]. Chronic morphine use can cause immunosuppression, and morphine addicts often have an increased incidence of viral hepatitis, bacterial pneumonias, endocarditis, tuberculosis and CNS infections [11,12]. Conversely, chronic morphine exposure can also indirectly potentiate an immune response by desensitizing the HPA axis [9,13,14], and increasing the production and activity of various cytokines, including IL-1β [15], IL-6 [16], and TNF-α [14]. Early studies showed that IL-1 increases the expression of opioid receptors in primary human glial cells [17] and human brain microvascular primary cells [3], suggesting that this pro-inflammatory cytokine may possess neuromodulatory effects and may be an important component in the immune-opioid circuit.

In this study, we hypothesized that a functional relationship may exist between IL-1β and the opioid receptors in human astrocytes. We examined the ability of IL-1β to increase expression of the MOR, DOR and KOR in a human astrocytic cell line, U87 MG, both in the untreated state as well as in the state desensitized with morphine. We then examined whether IL-1β-induced MOR up-regulation is mediated through the IL-1β receptor.

## Methods

### Materials

Cell culture and TRIzol® reagents were obtained from GIBCO/Invitrogen (Carlsbad, CA, USA). Morphine, naloxone and 12-*o*-tetradecanoyl-phorbol-13-acetate (TPA) and all other reagents used for RNA extraction were obtained from Sigma (St. Louis, MO, USA). DAPI was obtained from Pierce (Rockford, IL, USA), and antibodies to the MOR were obtained from Chemicon (Rosemont, IL, USA). Interleukin-1 beta (IL-1β) and interleukin-1 receptor antagonist protein (IL-1RAP) were obtained from R& D Systems (Minneapolis, MN, USA).

### U87 MG cells

Human astrocytoma cells (U87 MG), which have been previously used to represent astrocytes in *in vitro* studies [18-21], were obtained from American Type Culture Collection (ATCC) (Rockville, MD, USA) and grown in DMEM containing 10% FBS and 1% penicillin/streptomycin sulfate in a humidified 5% CO_2_ at 37°C.

### HL-60 cells

Human promyelocytic leukemia (HL-60) cells were obtained from ATCC (Rockville, MD, USA) and cultured in RPMI-1640 medium supplemented with 20% FBS and 1% penicillin/streptomycin sulfate. Cells were maintained at 37°C in humidified 5% CO_2_. The HL-60 cells were induced to differentiate into macrophage/monocyte-like cells with TPA (16 nM TPA/0.1% EtOH in RPMI-1640 medium) for 4 d. The TPA-treated medium was changed every 48 h until the completion of differentiation.

### NMB cells

Human neuroblastoma cells (NMB cells) were a gift from Dr. Horace H. Loh (University of Minnesota, MN, USA). NMB cells were grown and maintained in RPMI-1640 medium containing 10% FBS. NMB cells were maintained in a humidified environment of 5% CO_2_ at 37°C.

### SH-SY5Y cells

Human neuroblastoma cells (SH-SY5Y cells) were a gift from Dr. Robert Ross (Fordham University, New York, NY, USA). SH-SY5Y cells were grown and maintained in a 1:1 mixture of Earle's Minimum Essential Medium, Ham's Nutrient Mixture F12 and 10% FBS with penicillin/streptomycin sulfate.

### IL-1β treatment

U87 MG cells (2 x 10^5^ cells/well) were treated with cell culture medium containing either vehicle (cell culture medium) or IL-1β (20 ng/mL or 40 ng/mL) for 0, 3, 6, 12, 24, or 48 h. The medium was aspirated and 1 mL TRIzol® was added to each well. The cells were then frozen and stored at −80°C for further analysis.

### Co-treatment with IL-1β and IL-1RAP

U87 MG cells (2 x 10^5^ cells/well) were treated with cell culture medium containing either vehicle (cell culture medium), IL-1β (20 ng/mL), IL-1RAP (400 ng/mL) + vehicle, IL-1RAP (400 ng/mL) + IL-1β (20 ng/mL), IL-1RAP (4,000 ng/mL) + vehicle, or IL-1RAP (4,000 ng/mL) + IL-1β (20 ng/mL). IL-1RAP concentrations (400 ng/mL and 4,000 ng/mL) exceeded the manufacturer’s recommendation of a 1:100 ratio of IL-1β to IL-1RAP needed for IL-1RAP to be effective. Cells were then incubated in 5% CO_2_ at 37°C for 12 h. The medium was aspirated and 1 mL TRIzol® was added to each well. The cells were then frozen and stored at −80°C for further analysis.

### Time course of morphine’s effects on the MOR

U87 MG cells (1.5 x 10^5^ cells/well) were treated with fresh cell culture medium containing either vehicle or 100 nM morphine. Cells were incubated in 5% CO_2_ at 37°C for 45 minutes, 3, 6, 12, 24, or 48 hours. The medium was aspirated and 1 mL TRIzol® was added to each well. The cells were then frozen and stored at −80°C for further analysis.

### Pre-treatment U87 MG cells with morphine, followed by IL-1β

U87 MG cells (1.5 x 10^5^ cells/well) were treated with cell culture medium containing vehicle (cell culture medium) or 100 nM morphine [22]. Cells were incubated in 5% CO_2_ at 37°C for 24 h. The medium was aspirated and the cells were treated with fresh medium containing either vehicle or IL-1β (20 ng/mL). Cells were incubated in 5% CO_2_ at 37°C for 12 h. The medium was aspirated and 1 mL TRIzol® was added to each well. The cells were then frozen and stored at −80°C for further analysis.

### RNA isolation

Total RNA was extracted using TRIzol® reagent according to the manufacturer’s instructions (Invitrogen, Grand Island, NY, USA). Each treatment was performed in triplicate. The RNA was dissolved in RNase-free DEPC water, and the concentration of each sample was determined at an optical density of 260 nm, 280 nm and 320 nm using an ND-1000 Spectrophotometer (NanoDrop Technologies, Inc., Wilmington, DE, USA).

### Forskolin-induced cAMP accumulation assay

To evaluate the functionality of the MOR expressed in the U87 MG cells, a forskolin-induced cAMP accumulation assay as described previously [23], was conducted in U87 MG cells with and without morphine treatment. Each sample was assayed in triplicate. Upon reaching appropriate confluency (approximately 60 to 80%), the medium was removed and replaced with medium containing 0.5 mM IBMX, a phosphodiesterase inhibitor that blocks the breakdown of cAMP. The cells were incubated in 5% CO_2_ at 37°C for 30 minutes. The medium was aspirated and the cells were treated with 0.5 mL fresh medium containing either vehicle, 75 μM forskolin, 10 μM morphine, 10 μM morphine + 75 μM forskolin, or 10 μM morphine + 75 μM forskolin + 10 μM naloxone (a MOR antagonist). The cells were returned to the incubator for 10 minutes at 37°C. The medium was removed and the cells were washed twice with 1X PBS, then lysed with 0.1 N HCl. The cell lysates were frozen at −20°C until intracellular cAMP levels were measured using a commercially available RIA kit (Amersham Biosciences, Inc., Piscataway, NJ, USA). Parallel studies were conducted using two opioid peptides with a high affinity for the MOR, endomorphin-1 (10 μM) or endomorphin-2 (10 μM), in place of morphine.

### Radioimmunoassay (RIA)

Intracellular cAMP levels were measured in the thawed cell lysates by radioimmunoassay using the manufacturer’s protocol (Amersham Biosciences, Inc.). Each sample was assayed in triplicate. The cell lysate (10 μl), antiserum (100 μl) and I^125^ cAMP (100 μl) were added to glass culture tubes, vigorously shaken for two minutes, and then incubated overnight at RT. Following the incubation, 250 μl of Amelex-M secondary antibody was added to each tube, and the samples were incubated at RT for 10 minutes. Samples were then centrifuged for 15 minutes at 1,500 rpm at RT. The supernatant was decanted from each glass tube into a radioactive labeled container. A Wallac Wizard Gamma Counter 1470 was used to determine the amount of radioactivity (PerkinElmer, Waltham, MA, USA).

### Immunofluorescent staining

U87 MG cells were plated in a slide chamber containing 500 μl of DMEM + 10% FBS per chamber until 60 to 80% confluent. The U87 MG cells were then fixed in a paraformaldehyde and formaldehyde solution. The cells were washed with 1X PBS with Ca/Mg and dried. Slides were then incubated with a primary antibody [rabbit anti-MOR (1:1,000)] overnight at 4°C. Slides were washed with PBS and incubated with goat anti-rabbit IgG- FITC (1:1,000) for 2 h at RT. The nuclei were stained with DAPI for 20 minutes at RT, then washed with PBS. Confocal microscopy was used to visualize the sample (Olympus FluoView™ 1000, Center Valley, PA, USA).

### Real time reverse transcription polymerase chain reaction (rt-RT-PCR)

Absolute quantitative (AQ-rt-RT-PCR) and relative (rt-RT-PCR) real time PCR was performed to determine MOR, KOR, DOR and GAPDH expression levels following methods described in our published study [24]. Briefly, one microgram of total RNA was reverse transcribed to synthesize the first-strand cDNA on a GeneAmp 2400 Thermocycler (PerkinElmer) using 200 units of M-MLV reverse transcriptase, 10 mM DTT, 0.5 mM dNTP, 1X RT buffer, 5 ng/mL random primer and DEPC H_2_O for a total reaction volume of 20 μl (Invitrogen). Thermal cycling conditions were 37°C for 60 minutes, followed by a 10-minute incubation period at 65°C. The reaction was then cooled on ice. The cDNA (2 μl) was then amplified using real time PCR in a 50 μl PCR master mix (1 μl cDNA, 25 μl of 2X TaqMan® universal master mix, 1 μl of each probe (200 nM), 1 μl of each primer (400 nM), and 22 μl of DEPC H_2_O) in an ABI Prism 7000® (Applied Biosystems, Foster City, CA, USA). The PCR primers for MOR amplification were: 5'-TACCGTGTGCTATGGACTGAT-3' (sense), 5’-ATGATGACGTAA ATG- TGAATG-3' (antisense) and 5'/56 FAM/ CTTGCGCCTCAAGAGTGTCCGCA/ 3BHQ_1/-3' (probe). The primer for KOR amplification were: 5'-CGTCTGCTAC- ACCCTGATGATC-3' (sense), 5'-CTCTCGGGAGCCAGAAAGG-3' (antisense), and 5/56-HEX/TGCGTCTCAAGAGCGTCCGGC/3BHQ_2/-3’ (probe). The primers for DOR amplification were: 5'-GCGGGAAAGCCAGTGACTC-3' (sense), 5'-TGCCCTGTTTAAGGACTCAGTTG-3' (antisense), and 5'/56-JOE/AGGAGAG- GAGCGGGACCTGTGGCT/3BHQ_1/-3' (probe). A GAPDH mRNA fragment was also amplified for normalization of the MOR mRNA levels as previously described (Lai *et al.*, 2003) with the following sequence of primers and TaqMan probe: 5'-GGAAGCTCACTGGCATGGC-3' (sense), 5'-TAGACGGCAGGTCA- GGTCCA-3' (antisense) and 5'/56-FAM/CCCCACTGCCAACGTGTCAGTG/ 3BHQ_1/-3' (probe). The conditions for thermal cycling were as follows: 95°C for 10 minutes, followed by 40 cycles at 95°C for 18 s, then at 60°C for 1 minute. Analysis of the results was performed using the ABI Prism 7000® Sequence Detection System from Applied Biosystems.

### PCR data analysis

PCR data were analyzed based either on copy number or comparative C_T_ values (ΔΔCT). Copy number was determined using the standard curve method to determine copy number of the selected mRNA [24]. Briefly, the ABI PRISM 7000 Sequence Detection System software was utilized to determine the standard curve of the amplification of the appropriate sample using the appropriate standards (MOR, KOR, DOR and so on). The mean copy number was then determined for each treatment group, and the copy number of each sample was then normalized against a housekeeping control (GAPDH).

The comparative C_T_ method is a method used to determine threshold cycles (C_T_)_,_ the number of cycles that the fluorescence generated crosses a threshold as described previously [25]. Briefly, the C_T_ values were generated from the ABI PRISM 7000 Sequence Detection System software, and mean C_T_ values were determined for each treatment group. The difference in C_T_ values (ΔC_T_) was then determined by subtracting the mean C_T_ of the test samples from the mean C_T_ of the reference RNA. The ΔC_T_ for the test sample was then subtracted from the ΔC_T_ for the control sample to generate a ΔΔC_T_ as follows: ΔΔC_T_ = ΔC_T_ (test sample) − ΔC_T_ (control). The ΔΔC_T_ measurements were used to calculate expression of the test sample relative to the control and normalized to the untreated control: relative expression (fold change) = 2^−ΔΔ*C*^_*T*_.

### Statistical analysis

Data are presented as the mean ± SE. Statistical data were analyzed using either a one-way ANOVA, paired or unpaired Student’s *t*-test, as appropriate. Statistical significance was considered at *P* <0.05.

## Results

### Basal opioid receptor expression in immune and neuronal cell lines

**Figure 1:**
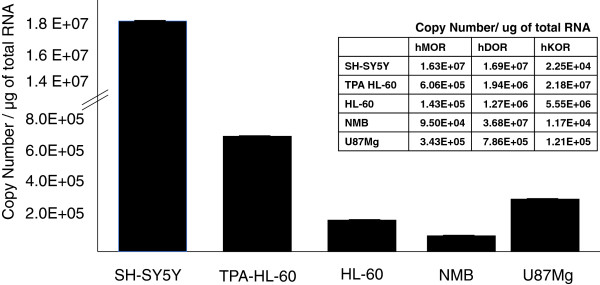
**Basal levels of MOR**, **DOR and KOR mRNA in neuronal and immune cell lines.** Basal levels (copy number) of the human MOR, DOR and KOR in the U87 MG astrocytic, HL-60 (TPA differentiated and undifferentiated), NMB, and SH-SY5Y cell lines were determined using absolute quantitative real time RT-PCR (AQ-rt-RT-PCR). GAPDH was used to normalize the levels in each cell line. Data are indicated as the mean ± SE.

Opioid receptor expression in the U87 MG astrocytoma cell line was compared to TPA-differentiated and undifferentiated HL-60 immune cells and SH-SY5Y and NMB neuronal cells. All of the cell lines examined expressed all three opioid receptors - MOR, DOR and KOR - although the levels of expression of each receptor varied in different cell lines. SH-SY5Y cells had the highest MOR expression (1.63 x 10^7^ ± 0.41 x 10^7^ copies of MOR/μg of total RNA) compared to the TPA-differentiated HL-60 cells (6.06 x 10^5^ ± 0.30 x 10^5^ copies of MOR/μg of total RNA), undifferentiated HL-60 cells (1.43 x 10^5^ ± 0.67 x 10^5^ copies of MOR/μg of total RNA), NMB cells (9.5 x 10^4^ ± 4.8 x 10^4^ copies of MOR/μg of total RNA), and U87 MG cells (3.43 x 10^5^ ± 0.67 x 10^5^ copies of MOR/μg of total RNA). NMB cells expressed the highest levels of DOR (3.68 x 10^7^ ± 0.48 x 10^7^ copies of DOR/μg of total RNA) compared to the TPA-differentiated HL-60 cells (1.94 x 10^6^ ± 0.3 x 10^6^ copies of DOR/μg of total RNA), undifferentiated HL-60 cells (1.26 x 10^6^ ± 0.06 x 10^6^ copies of DOR/μg of total RNA), SH-SY5Y cells (1.67 x 10^7^ ± 0.41 x 10^7^ copies of DOR/μg of total RNA), and U87 MG cells (7.86 x 10^5^ ± 0.06 x 10^5^ copies of DOR/μg of total RNA). TPA-differentiated HL-60 cells had the highest KOR expression (2.18 x 10^7^ ± 0.30 x 10^7^ copies of KOR/μg of total RNA) compared to HL-60 cells (5.55 x 10^6^ ± 0.14 x 10^6^ copies of DOR/μg of total RNA), NMB cells (1.17 x 10^7^ ± 0.48 x 10^7^ copies of DOR/μg of total RNA), SH-SY5Y cells (2.25 x 10^4^ ± 0.32 x 10^4^ copies of DOR/μg of total RNA), and U87 MG cells (1.21 x 10^5^ ± 0.13 x 10^5^ copies of DOR/μg of total RNA) (Figure 1).

### Immunofluorescence staining of the MOR in U87 MG cells

**Figure 2:**
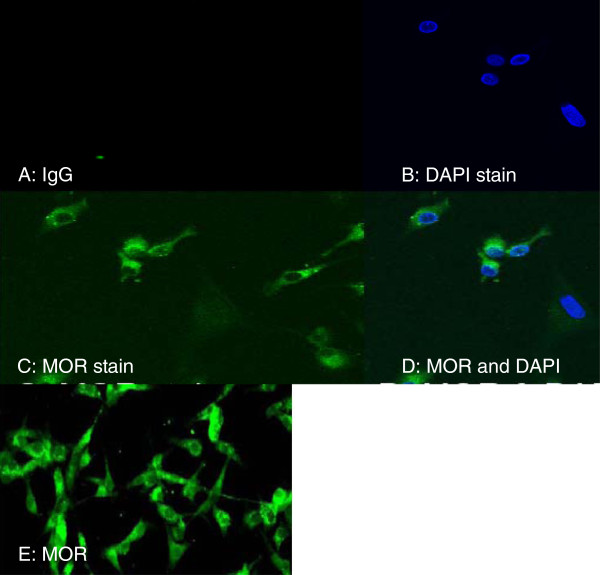
**Immunofluorescence staining of the human MOR.** Immunofluorescence staining was used to visualize the basal expression level of the human MOR. (**A**) U87 MG cells stained with goat anti-rabbit IgG (1:1,000) alone (negative control); (**B**) U87 MG cells stained with 4'-6-diamidino-2-phenylindole (DAPI) (1:1,000); (**C** and **E**) U87 MG cells stained with rabbit anti-MOR (1:1,000); (**D**) A superimposed image of B and C to show location of the MOR.

Immunofluorescence staining was used to further characterize the basal expression of the MOR in the U87 MG cells. U87 MG cells stained with goat anti-rabbit IgG alone served as a negative control (Figure 2A). DAPI staining was used to stain the nucleus, as shown in blue (Figure 2B). Rabbit anti-MOR staining indicated basal levels of MOR expression in the U87 MG cells, as indicated in green (Figure 2C, E). A superimposed image (Figure 2D) indicates the position of the MOR relative to the nucleus.

### Functionality of the MOR expressed in the U87 MG cells

**Figure 3:**
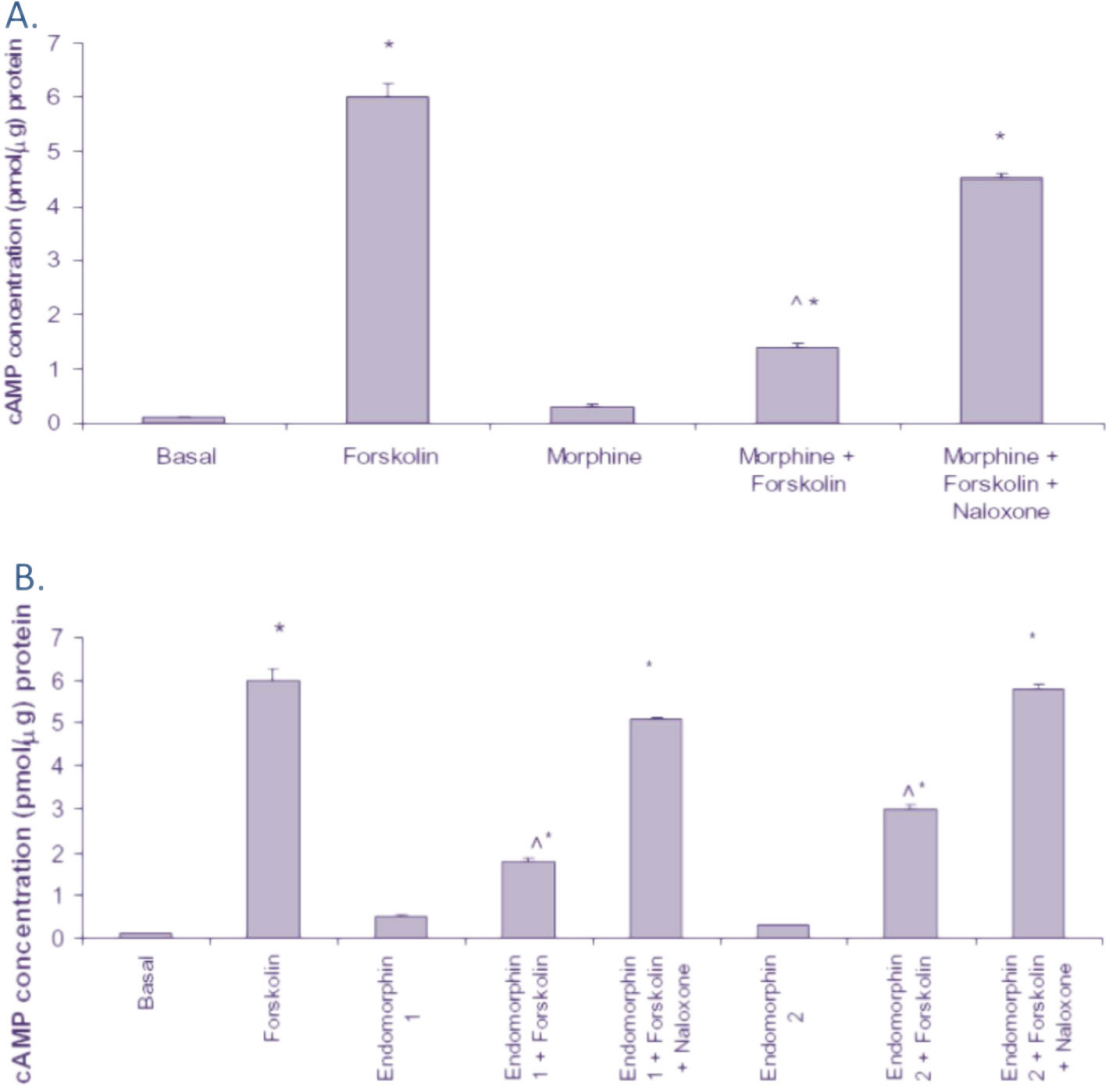
**The effects of morphine,naloxone**,**and endomorphin-1 and endomorphin-2,on forskolin-induced cAMP levels.** Functionality of the MOR expressed in U87 MG cells was determined using a forskolin-induced cAMP accumulation assay. (**A**) cAMP accumulation levels were determined in basal (untreated) U87 MG cells and in U87 MG cells treated with forskolin alone (75 μM), morphine alone (10 μM), forskolin (75 μM) + morphine (10 μM), or forskolin (75 μM) + morphine (10 μM) + naloxone (10 μM); (**B**) cAMP accumulation levels were determined in basal (untreated) U87 MG cells and in U87 MG cells treated with forskolin alone (75 μM), endomorphin-1 alone (10 μM), endomorphin-2 alone (10 μM), fosrkolin (75 μM) + endomorphin-1 (10 μM), forskolin (75 μM) + endomorphin-2 (10 μM), forskolin (75 μM) + endomorphin-1 (10 μM) + naloxone (10 μM), or forskolin (75 μM ) + endomorphin-2 (10 μM) + naloxone (10 μM). Data are the mean ± SE. A one-way ANOVA was used to determine significance. *P <0.05 compared to basal treatment; ^*P* <0.05 compared to forskolin treatment alone.

The MOR is a G protein-coupled receptor that, upon activation, initiates a signaling cascade that inhibits adenylyl cyclase, which in turn decreases intracellular cAMP levels [26]. In order to determine the functionality of the MOR expressed in the U87 MG cells, a forskolin-induced cAMP assay was conducted. As expected, forskolin (75 μM), an adenylyl cyclase activator, significantly increased cAMP levels (6.0 ± 0.25 pmol/μg protein) as compared to untreated control cells (0.1 ± 0.005 pmol/μg protein). U87 MG cells treated with morphine (10 μM) showed similar cAMP levels (0.3 ± 0.05 pmol/μg protein) as the control (0.1 ± 0.005 pmol/μg protein). U87 MG cells treated with morphine (10 μM) plus forskolin (75 μM) exhibited a significant decrease in cAMP levels (1.4 ± 0.075 pmol/μg protein) as compared to forskolin alone treated cells (6.0 ± 0.25 pmol/μg protein). The addition of a MOR antagonist, naloxone (10 μM), also increased cAMP levels (4.5 ± 0.075 pmol/μg protein) similar to forskolin (Figure 3A). Endomorphin-1 and endomorphin-2 are potent endogenous MOR agonists [27]. Treatment with endomorphin-1 or endomorphin-2 increased cAMP production similar to that observed with morphine treatment (Figure 3B).

### IL-1β-induced up-regulation of the MOR, DOR and KOR in U87 MG cells

**Figure 4:**
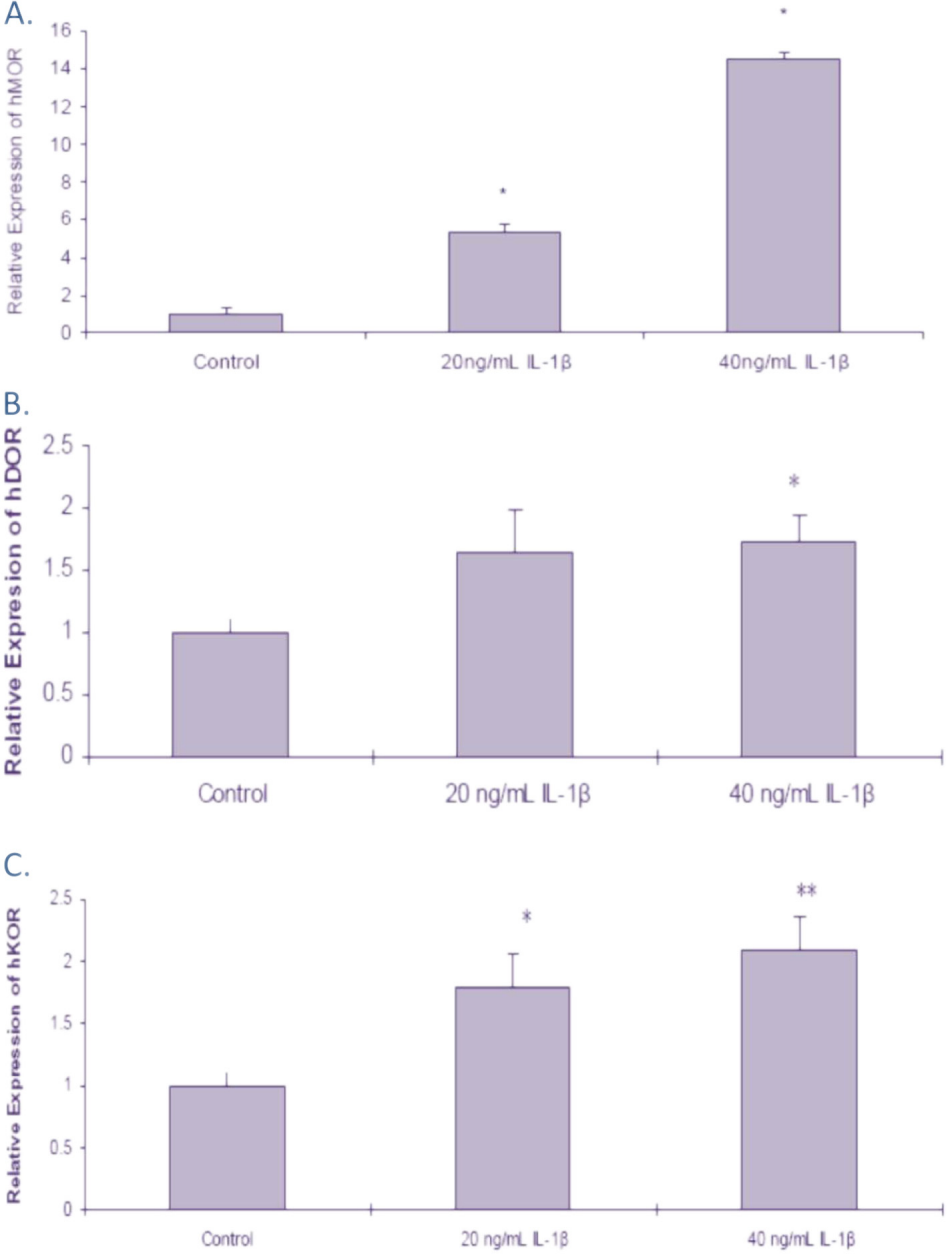
**The effects of IL-1βon opioid expression in U87 MG cells.** U87 MG cells were treated with either cell culture medium (control) or IL-1β (20 ng/mL or 40 ng/mL) for 12 h. Real time RT-PCR was used to determine the levels of MOR (**A**), DOR (**B**), and KOR (**C**); GAPDH was used to normalize the receptor levels. Data are the mean ± SE. A Student’s *t*-test was used to determine significance. *P <0.05 compared to control cells.

A significant increase in MOR expression was seen in a dose dependent manner in the U87 MG cells treated with IL-1β at 20 ng/mL and 40 ng/mL for 12 h (5.35 ± 0.44 and 14.5 ± 0.32, respectively) compared to control (Figure 4A). U87 MG cells treated with 40 ng/mL, but not 20 ng/mL, IL-1β had a significant increase in DOR compared to control (1.74 ± 0.24) (Figure 4B). A significant increase in KOR was observed in a dose dependent manner with 20 ng/mL and 40 ng/mL IL-1β (1.79 ± 0.27 and 2.09 ± 0.27, respectively) compared to the control (Figure 4C).

### IL-1β up-regulation of MOR expression via the IL-1β receptor

**Figure 5:**
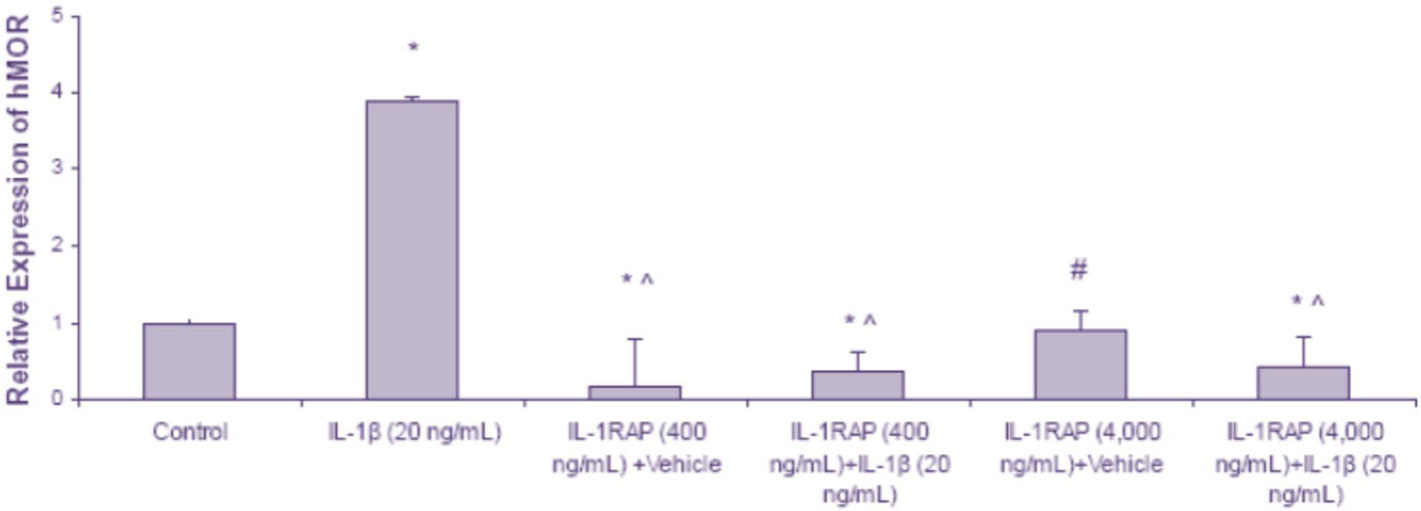
**The effects of IL-1RAP on IL-1β-induced up-regulation of the MOR in U87 MG cells.** U87 MG cells were treated with medium (control), IL-1β (20 ng/mL), IL-1RAP (400 ng/mL) + vehicle, IL-1RAP (400 ng/mL) + IL-1β (20 ng/mL), IL-1RAP (4,000 ng/mL) + vehicle, or IL-1RAP (4,000 ng/mL) + IL-1β (20 ng/mL) for 12 h. Real time RT-PCR was used to determine the levels of the MOR; GAPDH was used to normalize the MOR levels. Data are the mean ± SE. A Student *t*-test was used to determine significance. *P <0.05 compared to control; ^*P* <0.001 compared to IL-1β (alone); ^#^*P* <0.01 compared to IL-1β (alone).

IL-1β exerts its biological effects by binding to interleukin-1 receptor 1 (IL-1R1). An antagonist to this receptor, interleukin-1 receptor antagonist protein (IL-1RAP), has been shown to decrease IL-1β’s effects [28]. We used IL-1RAP to examine whether IL-1β’s up-regulation of the MOR is mediated through the IL-1R1. U87 MG cells treated with IL-1β (20 ng/mL) demonstrated an increase in the MOR (3.8 ± 0.05) compared to the U87 MG cells treated with control (Figure 5). U87 MG cells treated with IL-1RAP (400 ng/mL) + vehicle showed a significant decrease in MOR expression (0.16 ± 0.64) compared to IL-1β treatment alone or control. Co-treatment with IL-1RAP (400 ng/mL) + IL-1β (20 ng/mL) also resulted in a significant decrease of the MOR (0.36 ± 0.27) compared to the vehicle treated control and to IL-1β alone.

U87 MG cells treated with a higher concentration of IL-1RAP (4,000 ng/mL) + vehicle showed no difference in MOR expression (0.89 ± 0.24) compared to vehicle alone; however, there was a significant decrease compared to IL-1β alone (3.8 ± 0.05). The co-treatment with IL-1RAP (4,000 ng/mL) + IL-1β (20 ng/mL) resulted in a significant decrease in the MOR (0.43 ± 0.38) compared to the control and to IL-1β alone (Figure 5).

### The effects of morphine pre-treatment on IL-1β up-regulation of the MOR in U87 MG cells

**Figure 6:**
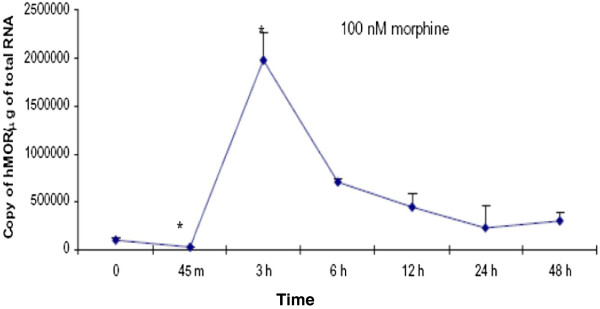
**The effects of morphine on MOR expression in U87 MG cells.** U87 MG cells were treated with either vehicle (cell culture medium) or morphine (100 nM) for 0 (control), 45 minutes, 3, 6, 12, 24, or 48 hours. Real time RT-PCR was used to determine the copy number of the MOR and GAPDH. GAPDH levels were used to normalize the MOR levels. Each time-point was adjusted by the appropriate time-point control. Data are the mean ± SE. A Student’s *t*-test was used to determine significance. *P <0.05 compared to control.

Chronic exposure to morphine desensitizes the MOR [29]. To further examine the immune-opioid relationship, IL-1β’s ability to potentially up-regulate a desensitized MOR after chronic morphine treatment was examined. A desensitization time course indicated that U87 MG cells treated with 100 nM morphine for 45 minutes had a significant decrease in the copy number of the MOR (2.5 x 10^4^ ± 3.7 x 10^3^ copies of MOR/μg total RNA) compared to the control (9.4 x 10^3^ ± 3.2 x 10^3^ copies of MOR/μg total RNA). Interestingly, after 3 h of morphine treatment, the MOR was significantly increased (1.9 x 10^6^ ± 2.9 x 10^4^ copies of MOR/μg total RNA) compared to the control. However, MOR expression again decreased after 6 h (7.0 x 10^5^ ± 2.9 x 10^4^ copies of MOR/μg total RNA), 12 h (4.4 x 10^5^ ± 1.4 x 10^4^ copies of MOR/μg total RNA), 24 h (2.2 x 10^5^ ± 2.3 x 10^4^ copies of MOR/μg total RNA), and 48 h (2.9 x 10^5^ ± 9.3 x 10^4^ copies of MOR/μg total RNA) of morphine treatment compared to control (Figure 6).

**Figure 7:**
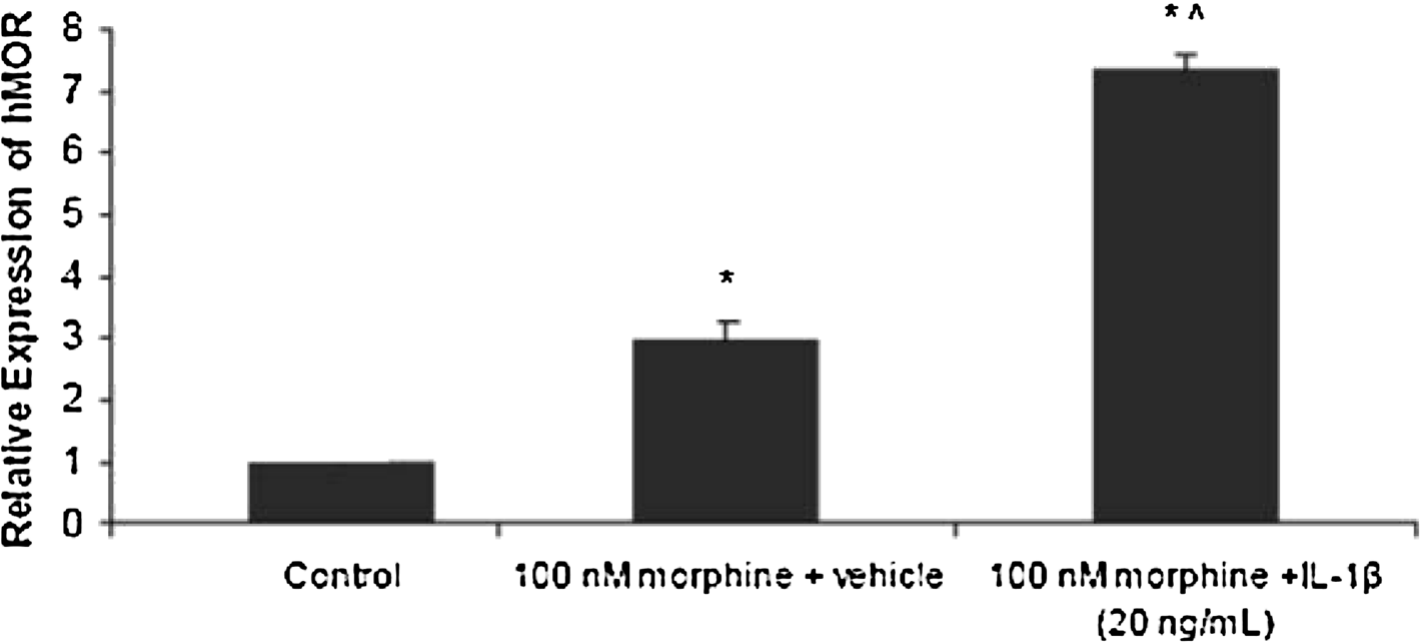
**The effects of morphine pre-treatment on IL-1β-induced up-regulation of the MOR in U87 MG cells.** U87 MG cells were pre-treated with either vehicle (control) or morphine (100 nM) for 24 h. Following pre-treatment, the medium was aspirated and fresh cell culture medium containing either vehicle or IL-1β (20 ng/mL) was added and the cells were incubated for 12 h. Absolute quantitative real time RT-PCR (AQ-rt-RT-PCR) was used to determine expression of the MOR and GAPDH. Levels of MOR were normalized to GAPDH. Data are the mean ± SE. A Student’s *t*-test was used to determine significance. *P <0.05 compared to the control; ^#^*P* <0.05 compared to IL-1β (alone); ^*P* <0.001 compared to the control.

In order to examine IL-1β’s ability to restore MOR levels after desensitization, U87 MG cells were treated with morphine (100 nM) for 24 h, followed by treatment with either vehicle or IL-1β (20 ng/mL) for 12 h. Cells treated with IL-1β alone exhibited similar MOR expression (0.9 ± 0.15) as the vehicle treated control (1.0 ± 0.8) (Figure 7). Cells treated with 100 nM morphine + vehicle showed a significant increase in MOR levels (2.94 ± 0.35) compared to the control. Cells treated with 100 nM morphine, followed by 20 ng/mL IL-1β for 12 h significantly increased MOR expression (7.32 ± 0.24) compared to the control as well as compared to cells treated with 100 nM morphine + vehicle.

## Discussion

In this study, we examined (a) the change in MOR expression in U87 MG human astrocytes exposed to IL-1β; (b) the ability of IL-1β to up-regulate the MOR desensitized by chronic morphine exposure; and (c) the role of the IL-1 receptor in IL-1β-mediated up-regulation of the MOR. Prior work in our laboratory showed that there is an alteration in LPS-induced cytokine expression when an animal is in a morphine tolerant state [14,30,31].

U87 MG cells are known to possess IL-1R1, the functional receptor for the IL-1 pro-inflammatory cytokine family [32]; however, the opioid receptors in this cell line have not yet been characterized. Using real time RT-PCR, we first determined the basal levels of the opioid receptors in the U87 MG cells. Our results showed that U87 MG cells exhibit moderate basal expression of all three receptors: MOR, DOR and KOR. We then verified that MOR is functionally active in the U87MG cells. MOR is a G-protein coupled receptor (GPCR) and inhibits adenylyl cyclase, which in turn decreases intracellular cAMP levels [33]. As a positive control, forskolin, which is known to activate adenylyl cyclase and increase cAMP levels, was used [34]. We also used morphine and other MOR agonists, such as endomorphin 1 and 2, as well as a MOR antagonist, naloxone. Our results show that the morphine and endomorphins were able to decrease the cAMP levels, whereas naloxone reverses the inhibitory effect, as we had expected. This proved that the MOR is functionally active in U87 MG cells and, therefore, is a good cell model to study the MOR signaling and activity.

We then examined IL-1β’s ability to modulate the expression of the opioid receptors. IL-1β induced a significant increase in MOR, DOR, and KOR, which is consistent with prior reports that IL-1 can induce MOR expression in human neural microvascular endothelial cells [3]. Although all three types of opioid receptors were significantly up-regulated by IL-1β, the increase in MOR was much more pronounced than DOR or KOR, suggesting that the MOR is a more potent mediator of an immune-opioid relationship than the DOR or KOR. Mohan *et al.* (2010) reported a similar observation in SK-N-SH neuroblastoma cells where MOR expression was increased after treatment with IL-1β. Since IL-1 cytokines are known to increase transcription through several different pathways, such as mitogen-activated protein kinases (MAPKs) and nuclear factor-κB (NF-κB), this could, in part, explain the increase in the opioid receptor transcription that is observed after IL-1β treatment [35,36].

The ability of morphine to desensitize opioid receptors is well established, as is the ability of IL-1β to up-regulate the MOR in neural microvascular endothelial cells, neuronal cells and glial cells [3,17,29]. In this study, we examined the ability of IL-1β to affect desensitized MOR in U87 MG cells. A time course revealed that MOR expression increased significantly after 3 h of morphine exposure, followed by a desensitization of the MOR. After 24 h of pretreatment with morphine, there was an increase in IL-1β-induced MOR expression in U87 MG as compared to U87 MG cells not pretreated with morphine. This was potentially due to a rebound effect which occurred when the morphine-containing medium was aspirated and replaced with non-morphine containing medium. The removal of the morphine-enriched medium could have precipitated cellular morphine withdrawal, which can cause MOR up-regulation [6]. However, morphine-desensitized U87 MG cells treated with IL-1β showed a significant increase in the MOR as compared to morphine treated cells not exposed to IL-1β. These data indicate that pro-inflammatory cytokines are able to modulate opioid receptors, both in an untreated and a morphine desensitized state in astrocytes. Pro-inflammatory cytokines, such as IL-1β, can affect the opioid-dependent pathways by stimulating the expression of the opioid receptors. Conversely, opioids, such as morphine, can induce the production of inflammatory cytokines. In rodent models, IL-1β expression was increased after administration of chronic morphine [37]. In another study, morphine was found to activate the toll-like receptor 4 (TLR4) in addition to the MOR. TLR4 is present on immune cells, such as microglia, which, upon activation, release pro-inflammatory cytokines, such as IL-1 [38]. Similar observations were reported in endothelial and neuroblastoma cells where morphine and IL-1β co-treatment increased MOR expression, which further substantiated our data [15,35].

IL-1RAP, an antagonist to the IL-1 receptor, was then used to determine the role of the IL-1β receptor in the modulation of the MOR. U87 MG cells treated with a high ratio of IL-1β to IL-1RAP (1:20) showed a significant decrease in MOR expression, whereas U87 MG cells treated with a low ratio of IL-1βto IL-1RAP (1:200) showed no difference in MOR expression compared to the control (only vehicle); however, there was a significant decrease in IL-1β-induced MOR expression compared to cells treated with only IL-1β. This indicates that the effect of IL-1β on MOR expression in U87 MG cells occurs through IL-1R1. The inability of the higher concentration of IL-1RAP to completely block IL-1β-induced up-regulation of the MOR expression could be because of over-saturation of the U87 MG cells. Another reason could be that high concentrations of IL-1RAP (without co-treatment of IL-1β) can decrease basal levels of IL-1β to such an extent that there is a negative feedback mechanism. Shavit *et al.* reported that acute administration of IL-1RAP in mice immediately after termination of morphine-induced analgesia, resulted in an induction of analgesia suggesting the MOR was probably up-regulated and, hence, analgesia was reinstated [39].

Since astrocytes co-exist with neuronal cells in the nervous system, it is important to take into account the effect of one on the other. It has been seen that lipopolysaccharide (LPS) stimulates MOR expression in both neuronal and macrophage-like cell models, through accumulation of reactive oxygen species (ROS) and pro-inflammatory cytokines [40], leading to further immune suppression and, thus, bringing about homeostasis. This suggests that IL-1β not only stimulates MOR expression in astrocytes but also in neuronal and other immune cells, highlighting the involvement of opioid receptors in the neuroimmune axis.

Our data also simulate the conditions seen in Human Immunodeficiency Virus 1 (HIV-1) infection, where IL-1β expression is up-regulated through activation of the NLAP3 inflammasome [41]. Since the derogatory effects of opioids on HIV-1 infection have been widely reported, it is possible that the HIV-1 induced increase in IL-1β secretion can also increase MOR expression in astrocytes, as suggested by our data.

## Conclusion

Our findings showed that IL-1β can increase the expression of the MOR, DOR and KOR in a human astrocytic cell line, U87 MG, both in the untreated state as well as in a state desensitized with morphine. This modulation is mediated through the IL-1β receptor. This suggests that, upon exposure to an inflammatory stimulus, activation of pro-inflammatory cytokines, such as IL-1β, occurs, stimulating the expression of the MOR, which could further suppress the inflammatory response and restore homeostasis.

## Abbreviations

ΔΔCT: Comparative C_T_ values; ATCC: American Type Culture Collection; AQ-rt-RT-PCR: Absolute quantitative reverse transcriptase real-time polymerase chain reaction; ANOVA: Analysis of variance; cAMP: Cyclic adenosine monophosphate; CNS: Central nervous system; DEPC: Diethylpyrocarbonate; DOR: Delta opioid receptor; GAPDH: Glyceraldehyde 3-phosphate dehydrogenase; GPCR: G-protein coupled receptor; HIV-1: Human immunodeficiency virus 1; HPA: Hypothalamic-pituitary-adrenal; IL-1: Interleukin-1; IL-1α: Interleukin 1, alpha; IL-1β: Interleukin 1, beta; IL-6: Interleukin 6; IL-RAP: Interleukin 1 receptor antagonist; KOR: Kappa opioid receptor; LPS: Lipopolysaccharide; MAPKs: Mitogen-activated protein kinases; MMLV: Moloney Murine Leukemia Virus; MOR: Mu opioid receptor; NALP3: NACHT, LRR and PYD domains-containing protein 3; NF-κB: Nuclear factor-κB; RIA: Radioimmunoassay; RT: Room temperature; rt-RT-PCR: Real-time polymerase chain reaction; TNFα: Tumor necrosis factor alpha; TLR4: Toll-like receptor 4.

## Competing interests

There are no competing interests.

## Authors’ contributions

LSB performed experiments and participated in the design of the study and drafting of the manuscript. JP carried out the experiments and performed the statistical analysis. SS participated in drafting the manuscript. SLC designed the studies, participated in its coordination and drafted the manuscript. All authors read and approved the final manuscript.
